# Stability analysis of the effect of harmonic waves on the shape stability of acoustic cavitation bubbles^☆^

**DOI:** 10.1016/j.ultsonch.2025.107444

**Published:** 2025-06-24

**Authors:** Kanji D. Hattori, Takuya Yamamoto

**Affiliations:** Department of Chemical Engineering, Graduate School of Engineering, Osaka Metropolitan University, 1-1, Gakuen-cho, Naka-ku, Sakai, Osaka 599-8531, Japan

**Keywords:** Stability analysis, Acoustic cavitation, Harmonic waves, Keller–Miksis equation

## Abstract

A liquid irradiated with high-power ultrasound generates harmonics and broad-band noise. The effect of ultrasound-containing harmonics on the bubble shape stability was numerically investigated in this study by solving the Keller-Miksis equation and the dynamic equation of distortion amplitude to identify the effect of harmonics on the equilibrium bubble size of acoustic cavitation. Numerical results indicated that the tendency of bubble stability changed for low- and high-pressure amplitudes, which could be attributed to decrease in the bubble expansion because of harmonic waves at high-pressure amplitude. The cavitation bubble became more unstable under low-pressure amplitude conditions, and more stable at high-pressure amplitude conditions because of harmonics; however, the discrepancy in the stable zones was small. The bubble wall velocity contributed to bubble stability under both amplitude conditions. The magnitude of the maximum bubble wall velocity increased with harmonic waves for the small pressure-amplitude conditions, resulting in an unstable bubble shape. The time that the bubble wall spent moving at high velocity was shortened with increasing harmonic waves under the high-pressure amplitude conditions, causing a stable bubble shape. Further, the phase shift of the harmonic wave affected bubble stability; bubble expansion was largely suppressed, and the bubble became more stable with an increase in the minimum pressure of the composite wave.

## 1 Introduction

Acoustic cavitation, which occurs when an aqueous solution is irradiated with ultrasound, leads to many phenomena [[1], [2], [3]]. For example, acoustic cavitation causes the inner temperature of the bubble to exceed 5000 K at the moment of bubble collapse, and the molecules in the bubble are thermally decomposed into some radicals, resulting in sonochemical reactions in the aqueous solution [4]. Cavitation bubbles oscillate at a high rate and form a micro-jet under an asymmetric condition [[5], [6], [7], [8], [9]]. Further, acoustic streaming is also developed in an ultrasonic bath because of cavitation motion and the dissipation of acoustic power [[10], [11], [12]]. Capillary waves are formed at a gas–liquid or liquid–liquid interface. These phenomena have been widely used in many applications, including organic chemical decomposition [13,14], emulsification [[15], [16], [17]], atomization [18], and extraction [19,20].

**Fig. 1:**
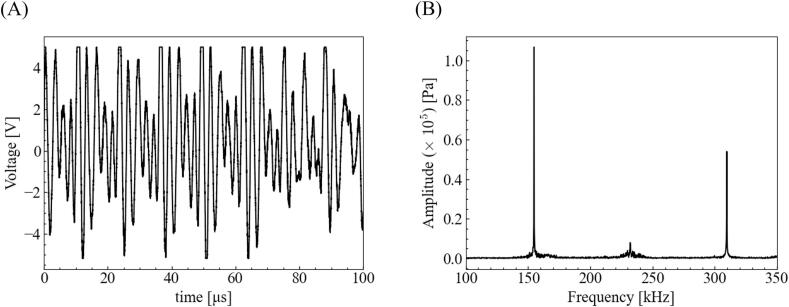
Acoustic signals obtained by sound pressure measurement using a hydrophone: (A) time variation of the sound pressure in an ultrasonic bath when the ultrasonic power is 84 W. (B) Pressure amplitude for each frequency obtained using the fast Fourier Transform of sound pressure.

Despite the development of the abovementioned applications, many phenomena that occur simultaneously in an ultrasonic bath are yet to be investigated because measuring them simultaneously is difficult. One such unclear phenomenon is the generation of harmonic waves. For example, consider the time variation of a sound wave measured using a hydrophone. The time variation of the sound pressure measured using a hydrophone and the fast Fourier transform (FFT) results for this signal are illustrated in Fig. 1, and the experimental data are obtained from our previous study [21]. As indicated in Fig. 1 and reported previously [2,[22], [23], [24], [25], [26], [27], [28], [29], [30], [31], [32]], a harmonic wave composition is generated in the measured ultrasonic wave. Thus far, several harmonic or subharmonic wave generation mechanisms have been proposed, including shockwaves [31,32], chaotic bubble motion [26,33], bubble–bubble interaction [24,[28], [29], [30]], nonspherical bubble deformation [34] and nonlinear wave propagation [23,27]. Although no single mechanism has been determined to be correct, a need exists to determine if the equilibrium bubble diameter in acoustic cavitation changes when a harmonic wave is generated. To the best of our knowledge, no previous study has discussed the effect of a generated harmonic wave on the equilibrium bubble size and bubble stability.

In this study, we investigated the effect of generated harmonic waves on bubble stability and equilibrium bubble size via a stability analysis of the shape of an acoustic cavitation bubble by solving the Keller–Miksis equation and dynamic equation for distortion amplitude. We investigated the effects of harmonics and their phase shifts on the shape stability of acoustic bubbles, assuming that sound waves generated during high-power ultrasound irradiation were superimposed composite waves between the fundamental and period-doubling ultrasound.

## 2 Numerical analysis

Bubble stability was evaluated by solving (i) the Keller–Miksis equation and (ii) the dynamic equation for small distortions. The Keller–Miksis equation predicts the nonlinear oscillations of spherical cavitation bubbles, whereas the dynamic equation for small distortions predicts the time variation in the distortion amplitude of spherical harmonics with respect to the bubble surface shape. These equations are solved simultaneously to calculate the time variations in the bubble radius and distortion amplitude. Harmonic waves are modeled by a composite wave between the ultrasonic fundamental and second-harmonic waves. The bubble stability was evaluated based on distortion growth for the second ultrasonic cycle to eliminate the influence of initial condition on the results. In this study, we assumed that acoustic bubbles cannot exist stably in the unstable zone because bubbles in this zone are easily fragmented [35,36].

### 2.1 Keller–Miksis equation

The expansion and contraction of a spherical bubble were modeled using the Keller–Miksis equation [1,37]

(1) $$\left(1-\frac{\dot{R}}{c_{\infty }}\right)R\ddot{R}+\frac{3}{2}{\dot{R}}^{2}\left(1-\frac{\dot{R}}{c_{\infty }}\right)=\frac{1}{\rho_{L,\infty }}\left(1+\frac{\dot{R}}{c_{\infty }}\right)\left[p_{B}-p_{s}\left(t\right)-p_{0}\right]+\frac{R}{c_{\infty }\rho_{L,\infty }}\frac{dp_{B}}{\mathrm{dt}},$$

where *R* is the bubble radius, *c*_∞_ is the speed of sound, *ρ*_L∞_ is the liquid density, *p*_B_ is the liquid pressure at the bubble wall, *p*_s_ is the sinusoidal ultrasonic pressure, *p*_0_ is the atmosphere pressure, and *t* is time. The liquid pressure at the bubble wall *p*_B_ and imposed sinusoidal ultrasonic pressure *p*_s_ are described as

(2) $$p_{B}=p_{g}+p_{v}-\frac{2\sigma }{R}-\frac{4\mu \dot{R}}{R},$$

(3) $$p_{s}\left(t\right)=\alpha_{1}P\sin\left(-\omega_{1}t\right)+\alpha_{2}P\sin\left(-\omega_{2}t+\theta \right),$$

where *p*_g_ is the pressure inside the bubble, *p*_v_ is the vapor pressure, *σ* is the surface tension, *μ* is the dynamic viscosity, *P* is the pressure amplitude, *ω*_1_ is the fundamental angular frequency, *ω*_2_ is the second harmonic angular frequency, *θ* is the phase shift, and *α*_1_, *α*_2_ are the ratio of fundamental and second harmonic waves. We applied a composite ultrasonic wave to consider harmonic waves. The pressure inside the bubble *p*_g_ is modeled as

(4) $$p_{g}=\left(p_{0}+\frac{2\sigma }{R_{0}}-p_{v}\right){\left(\frac{R_{0}}{R}\right)}^{3\gamma },$$

where *γ* represents the specific heat ratio.

The ultrasonic power was kept constant even when the ratio of the harmonics differed to compare the numerical results under fair conditions. To satisfy this condition, the ratios *α*_1_ and *α*_2_ were set as

(5) $$\alpha_{1}^{2}+\alpha_{2}^{2}=1$$

The detailed derivation of this relationship is described in Appendix A.

In this numerical model, the simulation becomes unstable under high-power conditions because the bubble wall velocity becomes larger than the speed of sound in water, and the terms on the left-hand side of the Keller–Miksis equation become negative. Therefore, additional modeling is required to model the dynamic behavior of bubbles under high-power conditions. Shockwaves are observed in an actual experiment, and the bubble wall velocity becomes close to or slightly larger than the speed of sound in water. The bubble wall velocity was limited by the speed of sound in water at the bubble wall, *c*_L,B_ [38] to simplify the numerical model and improve numerical stability. *c*_L,B_ is modeled as

(6) $$c_{L,B}=\sqrt{\frac{7.15\left(p_{B}+B\right)}{\rho_{L,i}}}$$

where *B* is the pressure parameter, and *ρ*_L,i_ is the liquid density at the bubble wall [1,39].

The physical properties and calculation parameters used in this study are listed in Table 1. Air and water were used as the simulated fluids, and the sound pressure amplitude, equilibrium bubble radius, phase shift, and ratio of the fundamental ultrasonic waves were varied. The frequencies of the fundamental and second harmonic waves were the same as those used in our experiment [21].

**Table 1:** Physical properties and parameters used in this stability analysis.

Properties	Value	Unit
Liquid density *ρ*_L,∞_	9.97 × 10^2^	kgm^−3^
External pressure *p*_0_	1.00 × 10^5^	Pa
Liquid sound velocity *c*_∞_	1.50 × 10^3^	ms^−1^
Liquid kinematic viscosity *ν*	8.92 × 10^−7^	m^2^s^−1^
Surface tension *σ*	7.20 × 10^−2^	Nm^−1^
Ratio of specific heat *γ*	1.40	−
Pressure parameter *B*	3.05 × 10^8^	Pa
Sound pressure amplitude *P*	5.00–40.0 × 10^4^	Pa
Equilibrium bubble radius *R*_0_	0.0800–3.20 × 10^−6^	m
Fundamental angular frequency *ω*_1_	155000 × 2*π*	rads^−1^
Second harmonic angular frequency *ω*_2_	310000 × 2*π*	rads^−1^
Phase shift *θ*	−π–π/2	rads^−1^
Ratio of fundamental wave *α*_1_	0.7–0.9	−

### 2.2 Dynamic equation for small distortion

Shape instability was investigated based on the time variation of a small shape distortion that was presented initially. Numerical models used in this study are based on those proposed by Brenner et al. [40] and Hilgenfeldt et al. [41]. The following dynamic equation for small distortions is obtained by introducing the distortion of spherical harmonics into the bubble dynamic equation.

(7) $${\ddot{a}}_{n}+B_{n}\left(t\right){\dot{a}}_{n}-A_{n}\left(t\right)a_{n}=0$$

where *A*_n_ and *B*_n_ are model parameters, *a*_n_ is the distortion amplitude, and subscript *n* is the degree of spherical harmonics. Parameters, *A*_n_ and *B*_n_ are modeled as

(8) $$A_{n}\left(t\right)=\left(n-1\right)\frac{\ddot{R}}{R}-\frac{\beta_{n}\sigma }{\rho R^{3}}-\frac{2\nu \dot{R}}{R^{3}}\left[\left(n-1\right)\left(n+2\right)+2n\left(n+2\right)\left(n-1\right)\frac{\delta }{R}\right]$$

(9) $$B_{n}\left(t\right)=3\frac{\dot{R}}{R}+\frac{2\nu }{R^{2}}\left[\left(n+2\right)\left(2n+1\right)-2n{\left(n+2\right)}^{2}\frac{\delta }{R}\right]$$

where *β*_n_ is described as

(10) $$\beta_{n}=\left(n-1\right)\left(n+1\right)\left(n+2\right)$$

In this model, the thickness of the boundary layer formed along the bubble surface *δ* is modeled using a viscous diffusive length scale with a cut-off length [41].

(11) $$\delta =\min\left(\sqrt{\frac{\nu }{\omega_{1}}},\frac{R}{2n}\right)$$

Similar to that in the previous studies [40,[42], [43], [44], [45]], the stability threshold was determined based on the maximum amplitude of the distortion in an ultrasonic cycle and the growth rate of the distortion amplitude. In this stability analysis, the Rayleigh–Taylor, parametric, and afterbounce instabilities occurred simultaneously. The generation condition of the Rayleigh–Taylor or afterbounce instabilities was evaluated as [40,41]

(12) $$\max_{\left\{t^{\prime }|t<t^{\prime }<t+T\right\}}\left(\frac{\left|a_{n}\left(t^{\prime }\right)\right|}{R\left(t^{\prime }\right)}\right)\geq 1$$

where *T* is an ultrasonic cycle period and *t*’ is an arbitral time. In this study, we refer to this instability as the afterbounce instability. In addition to this instability, the parametric instability was evaluated using the maximal eigenvalue of the Floquet transition matrix *F*_n_(*t* + *T*), which is defined as [40,41]

(13) $$\left(\begin{array}{c} a_{n}\left(t+T\right)\\ {\dot{a}}_{n}\left(t+T\right) \end{array}\right)=F_{n}\left(t+T\right)\left(\begin{array}{c} a_{n}\left(t\right)\\ {\dot{a}}_{n}\left(t\right) \end{array}\right)$$

The degree of spherical harmonics affects stability. Although the effect of spherical harmonic degree on the instability was investigated, the most unstable degree in this study was 2. Therefore, in the subsequent sections, the instability was evaluated with *n* = 2, and the initial amplitude of distortion was set to 1 nm. As reported in the previous studies [42,46], the initial amplitude does not change the instability.

### 2.3 Numerical models

The Keller–Miksis equation and dynamic equation of small distortion were discretized using the fourth-order Runge–Kutta method. The program was validated and verified in our previous studies [42,43]. In these studies, we compared our simulated bubble dynamic behavior with experimental [47] and numerical results [48], and the effects of the discretization methods and the equation used on the dynamic motion of the bubble radius were carefully compared. Finally, we confirmed that our program accurately predicted the shape instability of acoustic cavitation.

To ensure that the simulation did not diverge and the temporal discretization error was negligibly small, the time step Δ*t* was set to 5.0 × 10^−4^ ns, and the simulation was continued for 2.0 cycles of ultrasound. Stable and unstable borders can be observed in the stability diagram when the pressure amplitude is close to or higher than the cavitation occurrence threshold, and therefore, the investigated parameter ranges for the equilibrium radius and pressure amplitude were 0.0800–3.20 μm, and 5.00–40.0 × 10^4^ Pa, respectively. The simulation was conducted at intervals of 6.0 × 10^−3^ μm for the equilibrium radius and 5.0 × 10^2^ Pa for the pressure amplitude. The spatial resolution of the obtained stability diagram was 521 × 701.

## 3 Numerical results

### 3.1 Bubble shape instability without phase shift

**Fig. 2:**
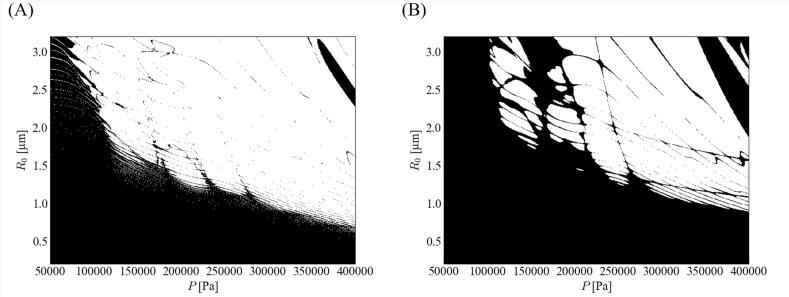
Stability diagrams of the (A) parametric instability and (B) afterbounce instability when the ratio of fundamental frequency *α*_1_ is 0.7. The stable zone is marked in black.

Fig. 2 shows the stability diagrams of the (A) parametric and (B) afterbounce instabilities when the ratio of the fundamental frequency *α*_1_ is 0.7. The bubble was stable for the small sizes and the low-pressure amplitudes in the same manner as reported in previous studies [[40], [41], [42], [43], [44],^46^,^49^]. In addition, the parametric instability was more severe than the afterbounce instability. Therefore, the bubble shape instability was determined using the parametric instability.

**Fig. 3:**
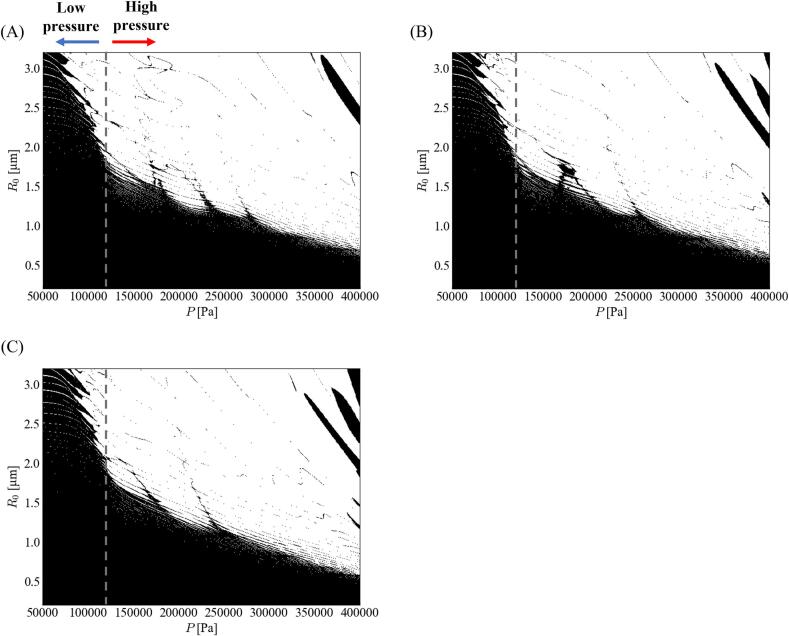
Stability diagrams of parametric instability with different ratios of fundamental frequency, *α*_1_: (A) 0.7, (B) 0.8, and (C) 0.9. The stable zone is marked in black color.

Fig. 3 shows the stability diagrams of the parametric instability for different ratios of the fundamental frequency. The tendency of this instability differed depending on the sound pressure amplitude. In Fig. 3, the threshold border (dotted line) was added to indicate where the tendency changed. This tendency changed at *P* = ∼120 kPa. We defined the steep- and gentle-slope zones as the low- and high-pressure amplitude zones, respectively. This phenomenon changes because of bubble oscillation around the threshold. At low-pressure amplitude, the bubble expansion was not weakened because of harmonic waves, although it was weakened at high-pressure amplitude. For a low-pressure amplitude, the stable zone shrinks slightly with a decrease in the ratio of the fundamental frequency, whereas the stable zone expands slightly with a decrease in the ratio of the fundamental frequency for a high-pressure amplitude.

**Fig. 4:**
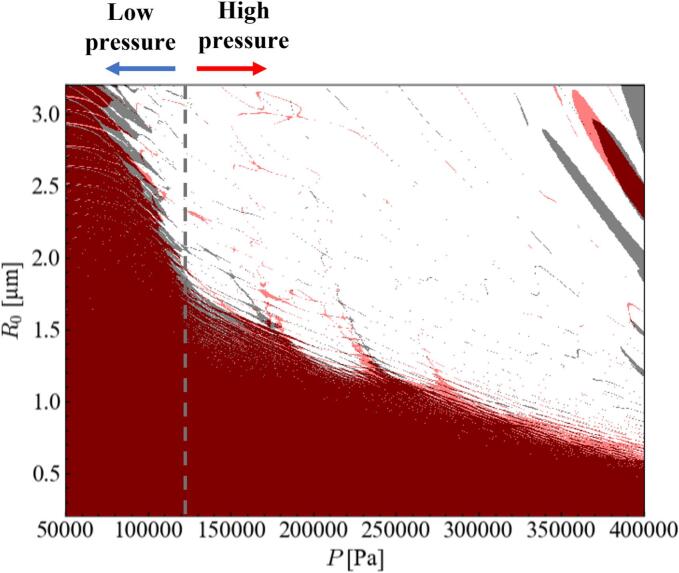
Differences in the stable zones between *α*_1_ = 0.9 (black) and 0.7 (red) in the stability diagram of parametric instability. (For interpretation of the references to color in this figure legend, the reader is referred to the web version of this article.).

Fig. 4 shows the difference in the stable zones between *α*_1_ = 0.9 and 0.7. The black and red colors were used for the stable zones for *α*_1_ = 0.9 and 0.7, respectively. The stable zone slightly shrinks and slightly expands for the low- and high-pressure amplitudes, respectively, with a decrease in the ratio of fundamental frequency.

**Fig. 5:**
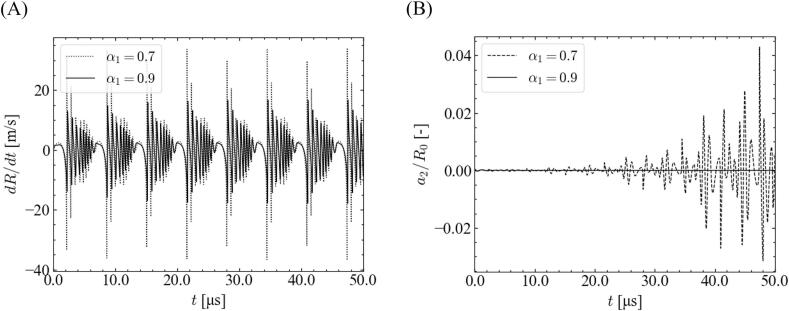
Time variations of the (A) bubble wall velocity and (B) normalized distortion amplitude with different ratios of fundamental frequency for *θ* = 0, *P* = 1.0 × 10^5^ Pa, and *R*_0_ = 2.5 μm.

Fig. 5 shows the time variations of the bubble wall velocity d*R*/d*t* and the normalized distortion amplitude *a*_2_/*R*_0_ with different fundamental frequency ratios. In this case, the equilibrium bubble radius and pressure amplitude are 2.5 μm and 1.0 × 10^5^ Pa, respectively. As shown in Fig. 5(A), the bubble wall velocity during the rebound period was higher for the smaller ratio of the fundamental frequency. Accordingly, the distortion amplitude gradually increases at a smaller ratio of the fundamental frequency, and the bubble shape becomes unstable. Thus, the stable zone shrinks with decreasing the ratio of fundamental frequency, as indicated in Fig. 3, Fig. 4.

**Fig. 6:**
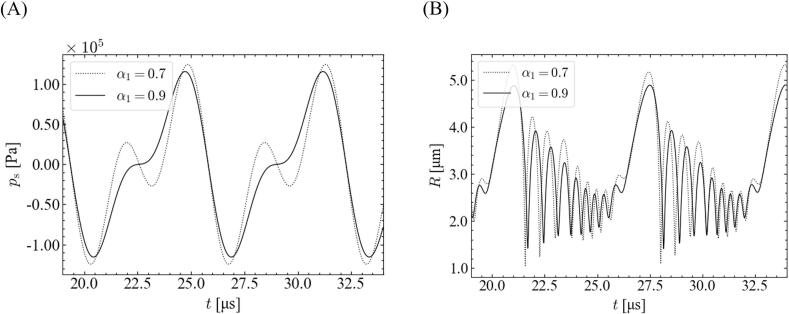
Time variations of (A) sound pressure and (B) bubble radius with different ratios of fundamental frequency for *θ* = 0, *P* = 1.0 × 10^5^ Pa, and *R*_0_ = 2.5 μm.

We focused on the relationship between the imposed ultrasonic pressure and bubble oscillation to investigate the origin of the discrepancy between *α*_1_ = 0.9 and 0.7. Fig. 6 shows the time variations in the sound pressure and bubble radius. The simulated conditions are the same as those shown in Fig. 5. The imposed pressure initially decreased largely, and the minimum pressure for ∼20–21 μs became smaller for *α*_1_ = 0.7; this smaller minimum imposed pressure caused a larger bubble expansion for ∼20–21.5 μs. The enlarged bubble caused a smaller bubble inner pressure, resulting in a violent collapse. Therefore, the bubble wall velocity increases at a lower ratio of the fundamental wave.

**Fig. 7:**
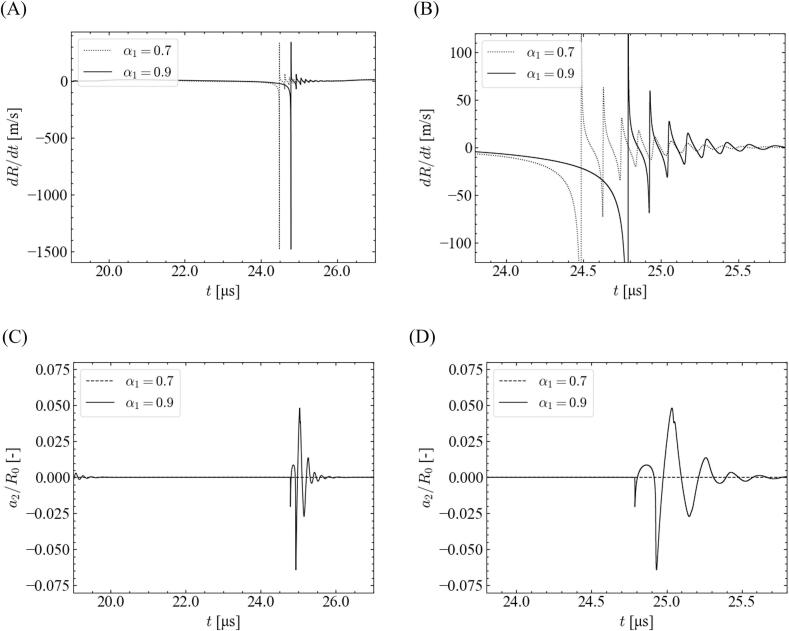
Time variations of (A, B) bubble wall velocity and (C, D) distortion amplitude with different ratios of fundamental frequency for *θ* = 0, *P* = 3.0 × 10^5^ Pa, and *R*_0_ = 1.0 μm. The time periods shown are (A, C) 19–27 μs, and (B, D) 23.8–25.8 μs.

As shown in Fig. 4, the stable zone for a high-pressure amplitude expanded with a decrease in the fundamental ratio, which can be explained in terms of time variations in the bubble wall velocity and distortion amplitude. Fig. 7 shows the time variations of the bubble wall velocity and distortion amplitude with different fundamental frequency ratios. In this case, the equilibrium bubble radius and pressure amplitude are 1.0 μm and 3.0 × 10^5^ Pa, respectively. Enlarged views of the rebound period are shown in (B) and (D). In this analysis, the bubble wall velocity is limited to ∼1500 m/s, which is the speed of sound in water. Therefore, as shown in Fig. 7(A) and (B), even though the ratios of the fundamental frequency are different, the magnitudes of the bubble wall velocities seem to be almost the same. However, during violent bubble collapse, the bubble wall velocity for *α*_1_ = 0.7 did not reach the speed of sound in water (1500 m/s), whereas that for *α*_1_ = 0.9 reached the speed of sound in water. Further, the time period when the magnitude of the bubble wall velocity reached 1480–1500 m/s during the violent collapse was slightly longer for *α*_1_ = 0.9 (8.0 × 10^−3^ ns), compared to that for *α*_1_ = 0.7 (5.0 × 10^−3^ ns). Therefore, the distortion amplitude increases at a larger ratio of the fundamental frequency as shown in Fig. 7(C) and (D), and the bubble shape becomes unstable.

**Fig. 8:**
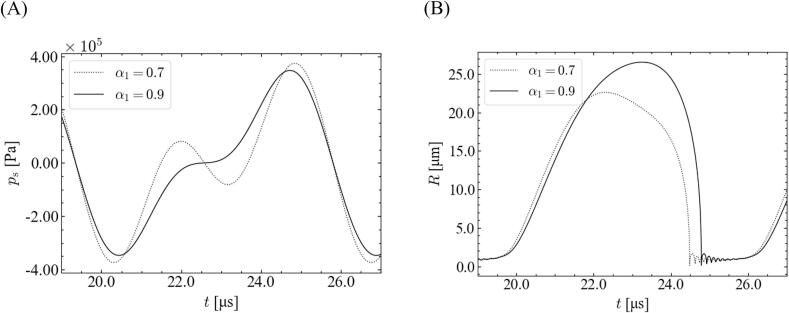
Time variations of (A) imposed sound pressure and (B) bubble radius with different ratios of fundamental frequency for *θ* = 0, *P* = 3.0 × 10^5^ Pa, and *R*_0_ = 1.0 μm.

We focused on the relationship between the imposed sound pressure and the bubble oscillation to investigate the origin of this discrepancy between *α*_1_ = 0.9 and 0.7. Fig. 8 shows the time variations of the imposed sound pressure and bubble radius for different fundamental frequency ratios. The simulated conditions are the same as those shown in Fig. 7. For the high-pressure amplitude, the bubble expansion was disturbed for *α*_1_ = 0.7 in 22–23.5 μs, and the maximum bubble size became smaller for *α*_1_ = 0.7. Accordingly, the bubble wall velocity becomes slightly lower for *α*_1_ = 0.7.

### 3.2 Bubble shape instability with phase shift

**Fig. 9:**
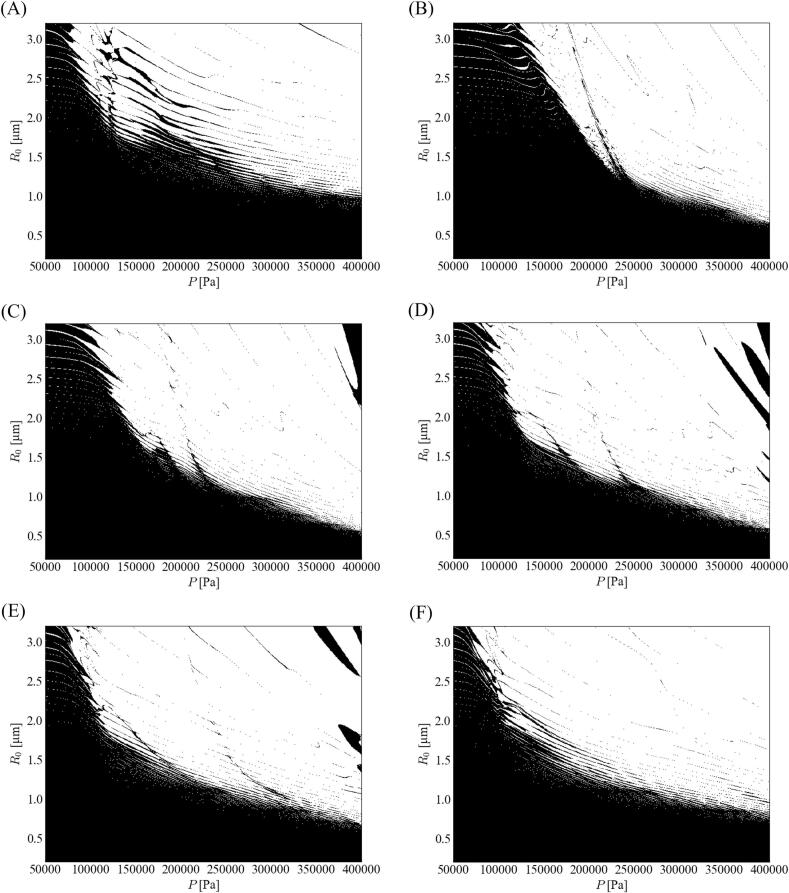
Stability diagrams of parametric instability for the case of *α*_1_ = 0.9 with different phase shifts: (A) *θ* = −*π*, (B) −*π*/2, (C) −*π*/4, (D) 0, (E) *π*/4, and (F) *π*/2.

We simulated the effect of phase shift on the bubble shape stability for different fundamental frequency ratios. The effect of the phase shift on the stability diagram was similar, even when the ratio of the fundamental frequency was different. Therefore, we only show the numerical results for the case of *α*_1_ = 0.9 as a representative value. Fig. 9 shows the stability diagrams of parametric instability for *α*_1_ = 0.9 with different phase shifts. The stable zone was the widest for *θ* = −*π*/2 and the bubble was the most stable for this condition. The stable zone shrinks, and the bubble shape becomes unstable as the phase shift deviates from −*π*/2.

**Fig. 10:**
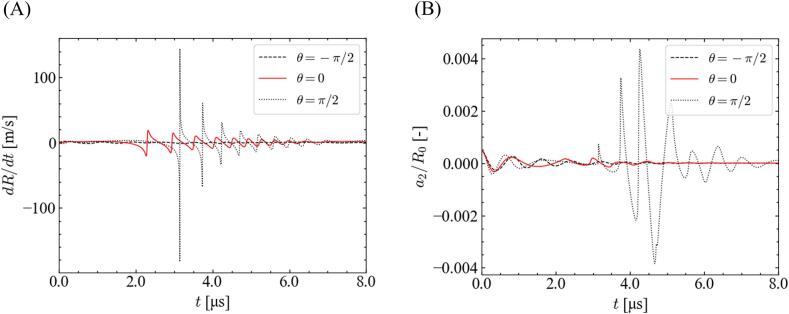
Time variations of the (A) bubble wall velocity and (B) normalized distortion amplitude with different phase shifts for *P* = 1.1 × 10^5^ Pa, *R*_0_ = 2.1 μm, and *α*_1_ = 0.9.

The reason why the bubble is the most stable for *θ* = −*π*/2 is discussed in terms of bubble oscillation. Fig. 10 shows the time variations of the bubble wall velocity and normalized distortion amplitude with different phase shifts. Under this condition, the pressure amplitude and equilibrium bubble radius are 1.1 × 10^5^ Pa and 2.1 μm, respectively. As shown in Fig. 10(A), the bubble wall velocity is the largest for *θ* = *π*/2, and it increases as the phase shift deviates from −*π*/2. Accordingly, the distortion amplitude increases for *θ* = *π*/2 when the bubble wall velocity is high. This change in stability caused by the phase shift can be explained by the bubble wall velocity.

**Fig. 11:**
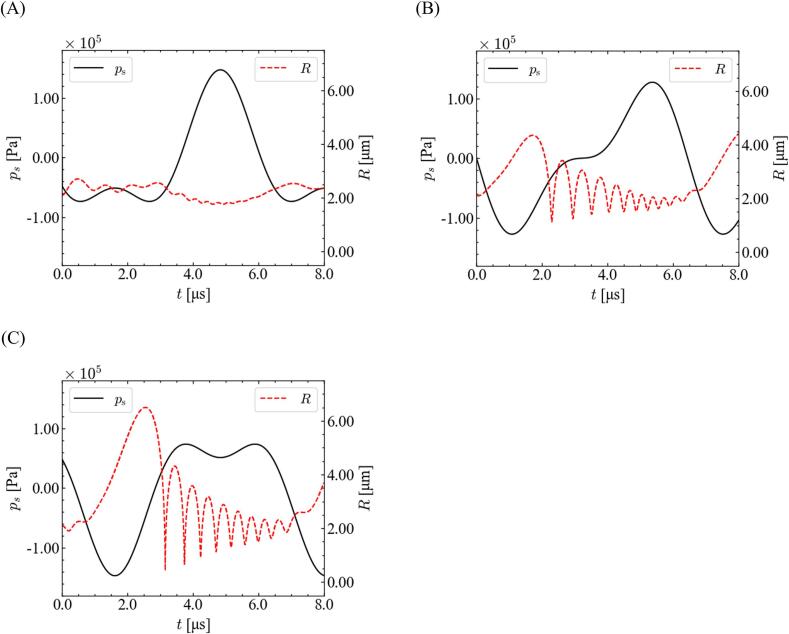
Time variations of the sound pressure and bubble radius for *P* = 1.1 × 10^5^ Pa, *R*_0_ = 2.1 μm, and *α*_1_ = 0.9 with different phase shifts: (A) *θ* = −*π*/2, (B) 0, and (C) *π*/2.

Fig. 11 shows the time variations in the sound pressure and bubble radius with different phase shifts. The simulated conditions are the same as those shown in Fig. 10. Bubble expansion was suppressed by harmonic waves when the phase shift deviated from *π*/2. The bubble expansion was suppressed largely for *θ* = −*π*/2 because the harmonic wave increased the minimum pressure of the fundamental ultrasonic wave, and therefore, the bubble became stable for *θ* = −*π*/2. This discussion is valid for the conditions of high-pressure amplitude and different ratios of fundamental waves.

## 4 Conclusions

In this study, we numerically investigated the effect of harmonic waves and their phase shifts on bubble shape instability by simultaneously solving the Keller–Miksis equation and the dynamic equation for distortion amplitude. The main findings of this study are summarized as follows:

• The tendency of bubble stability changed for low- and high-pressure amplitudes, and this phenomenon could be attributed to decrease in the bubble expansion because of harmonic waves at high-pressure amplitude.
• The bubble became more unstable under low-pressure amplitude conditions and more stable under high-pressure amplitude conditions because of harmonic waves. However, the discrepancy in the stable zone for the different harmonic wave ratios was small.
• For the low-pressure amplitude condition, the effect of the harmonic wave on the bubble shape instability could be explained by the change in the bubble wall velocity, which increased with decreasing in the fundamental frequency ratio, resulting in a more unstable bubble.
• For the high-pressure amplitude condition, the effect of the harmonic wave on the bubble shape instability could be explained by the maximum bubble expansion radius and the bubble wall velocity during a violent bubble collapse.
• The bubble was the most stable for *θ* = −*π*/2, and the stable zone shrank as the phase shift, *θ* deviates from −*π*/2 because the bubble expansion was most suppressed by the harmonic wave, and the bubble wall velocity became small for *θ* = −*π*/2.

## CRediT authorship contribution statement

**Kanji D. Hattori:** Writing – original draft, Visualization, Validation, Investigation, Formal analysis, Data curation. **Takuya Yamamoto:** Writing – review & editing, Validation, Software, Project administration, Methodology, Funding acquisition, Conceptualization.

## Declaration of competing interest

The authors declare that they have no known competing financial interests or personal relationships that could have appeared to influence the work reported in this paper.

## Appendix

### Appendix A Derivation of the constant ultrasonic power condition with different ratios of the harmonic wave

Ultrasonic power *P*_U_ is calculated for a single ultrasonic wave as

(A1) $$P_{U}=\frac{P^{2}A}{\rho_{L}c_{\infty }}$$

where *A* represents the surface area irradiated by ultrasound. Therefore, the ultrasonic power at two frequencies is calculated as

(A2) $$P_{U}=\frac{\left(P_{1}^{2}+P_{2}^{2}\right)A}{\rho_{L}c_{\infty }}$$

where *P_1_* is the pressure amplitude of fundamental wave, and *P_2_* is the pressure amplitude of harmonic wave. These pressure amplitudes are modeled using the ratios *α*_1_ and *α*_2_ as

(A3) $$P_{1}=\alpha_{1}P,$$

(A4) $$P_{2}=\alpha_{2}P,$$

Ultrasonic power with two frequencies must be the same as that with a single frequency to maintain constant ultrasonic power.

(A5) $$\frac{P^{2}A}{\rho_{L}c_{\infty }}=\frac{\left(P_{1}^{2}+P_{2}^{2}\right)A}{\rho_{L}c_{\infty }}.$$

The following relationship was obtained by substituting Eqs. (A3), (A4) into Eq. (A5):

(A6) $$\alpha_{1}^{2}+\alpha_{2}^{2}=1$$
